# Physical exercise prevents motor disorders and striatal oxidative imbalance after cerebral ischemia-reperfusion

**DOI:** 10.1590/1414-431X20154429

**Published:** 2015-07-28

**Authors:** P.M. Sosa, H.L. Schimidt, C. Altermann, A.S. Vieira, F.W.S. Cibin, F.P. Carpes, P.B. Mello-Carpes

**Affiliations:** 1 Grupo de Pesquisa em Fisiologia, Universidade Federal do Pampa, Uruguaiana, RS, Brasil; 2 Grupo de Pesquisa em Neuromecânica Aplicada, Universidade Federal do Pampa, Uruguaiana, RS, Brasil; 3 Laboratório de Biotecnologia da Reprodução, Universidade Federal do Pampa, Uruguaiana, RS, Brasil

**Keywords:** Stroke, Striatum, Locomotion, Oxidative stress, Antioxidants, Running

## Abstract

Stroke is the third most common cause of death worldwide, and most stroke survivors present some functional impairment. We assessed the striatal oxidative balance and motor alterations resulting from stroke in a rat model to investigate the neuroprotective role of physical exercise. Forty male Wistar rats were assigned to 4 groups: a) control, b) ischemia, c) physical exercise, and d) physical exercise and ischemia. Physical exercise was conducted using a treadmill for 8 weeks. Ischemia-reperfusion surgery involved transient bilateral occlusion of the common carotid arteries for 30 min. Neuromotor performance (open-field and rotarod performance tests) and pain sensitivity were evaluated beginning at 24 h after the surgery. Rats were euthanized and the corpora striata was removed for assay of reactive oxygen species, lipoperoxidation activity, and antioxidant markers. Ischemia-reperfusion caused changes in motor activity. The ischemia-induced alterations observed in the open-field test were fully reversed, and those observed in the rotarod test were partially reversed, by physical exercise. Pain sensitivity was similar among all groups. Levels of reactive oxygen species and lipoperoxidation increased after ischemia; physical exercise decreased reactive oxygen species levels. None of the treatments altered the levels of antioxidant markers. In summary, ischemia-reperfusion resulted in motor impairment and altered striatal oxidative balance in this animal model, but those changes were moderated by physical exercise.

## Introduction

Stroke is the third most common cause of death worldwide (1). Up to 20% of stroke survivors require long-term institutional care, and 15–30% are unable to perform daily life or work activities (2). Stroke events result from suppression of blood flow to the brain, which decreases oxygen and glucose delivery to brain tissue (3). This deprivation may result from disruption of a blood vessel, leading to hemorrhagic stroke, or from interruption of blood flow, leading to ischemic stroke (4). Most stroke events (85–90%) are ischemic in origin (5). In an ischemic event, blood reperfusion leads to tissue damage (6). Such damage has deleterious effects on important cellular structures including the basal membrane and mitochondria (7).

Evidence suggests that reperfusion injury results from oxidative stress (8) characterized by increased levels of reactive oxygen species (ROS) that induce neuronal damage due to lipid peroxidation (6). Under conditions of oxidative stress, cells are unable to balance the deleterious effects of ROS through antioxidant mechanisms (8). Some brain regions, including the striatum, appear to be particularly susceptible to oxidative damage due to ROS levels (9). The striatum plays an important role in the control of voluntary movements (10) and contains a high concentration of dopaminergic receptors, which are responsible for motor activation (11). Further, dopaminergic receptors are highly susceptible to ischemic damage (12). Therefore, in models of transient ischemia-reperfusion, rats can present motor impairments that may be explained by striatal damage resulting from oxidative stress and by neuronal death (13).

The high incidence of stroke, the disabilities observed among survivors (14), and the costs of currently available treatments have promoted efforts to improve post-stroke recovery and to prevent insults to the central nervous system. One interesting strategy is physical exercise, which is easy to offer to patients and does not involve high costs. It might thus become an important public health strategy. Previous reports suggest that physical exercise may be an effective neuroprotective strategy. Aerobic exercise ameliorates memory impairment after cerebral ischemia (12,15,16), reduces cognitive deficits related to aging (17), delays neurodegeneration in Alzheimer's disease models (18), and facilitates functional recovery after stroke (5). The mechanisms involved in these effects include the increase of antioxidant defenses in the hippocampus, promotion of neuronal resistance to oxidative stress (13), upregulation of BDNF (brain-derived neurotrophic factor) and VEGF (vascular endothelial growth factor) (19), and the prevention of neuronal death (1). In addition, acute exercise improves motor memory and skill acquisition (20).

Considering the results of previous studies, we assessed the neuroprotective role of physical exercise on the oxidative imbalance and motor impairments resulting from ischemia-reperfusion. Invasive experimental protocols cannot be conducted in humans, which makes animal experimentation important in advancing the understanding of behavioral and biochemical parameters associated with oxidative stress and allows dissection of brain structures. Thus, we used a rat ischemia-reperfusion model in the experiments described below.

## Material and Methods

### Animals and experimental groups

Forty male Wistar rats were purchased from the Central Vivarium of the Universidade Federal de Santa Maria (RS, Brazil) and housed 3 per cage under controlled light and environmental conditions (12-h light/dark cycle at 23±2°C and 50±10% humidity) with food and water *ad libitum*. All experiments were conducted in accordance with the National Institute of Health Guide for the Care and Use of Laboratory Animals (NIH, 1996) and the Animal Care and Use Committee (IRB #0132012) of the Universidade Federal do Pampa. The weight of each rat and the liquid ingested in each cage were measured daily. At the age of 2 months, rats were randomly assigned to one of four experimental groups. A control group (SHAM) was subjected to sham surgery without occlusion of the common carotid arteries. An ischemia-reperfusion group (ISCH) was subjected to surgery to produce temporary bilateral occlusion of the common carotid arteries. An exercise group (EXERC) performed physical exercise before sham surgery. An exercise and ischemia-reperfusion group (EXERC-ISCH) performed physical exercise before ischemia-reperfusion surgery.

**Figure 1:**
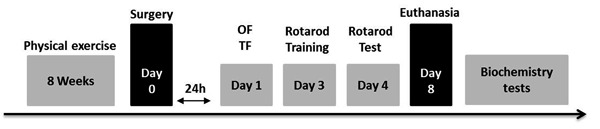
Rats in the exercise (EXERC) and exercise and ischemia-reperfusion groups (EXERC-ISCH) were subjected to 8 weeks of physical exercise on a treadmill for rodents. On day 0, rats from all groups underwent surgery with or without occlusion of the carotid arteries. Twenty-four hours after surgery rats were given open-field (OF) and tail-flick tests (TF). On the third day, rats were trained in the rotarod test and on the fourth day they were given the rotarod test. To avoid changes in brain markers resulting from stress due to the rotarod test, rats were euthanized and brain tissue was collected a few days later, on the eighth day post-surgery. Bilateral striatum tissue was removed and used in subsequent biochemical assays.

Rats were subjected to motor function testing beginning at 24 h after surgery, and 8 days after surgery. Rats were euthanized to collect brain tissue for evaluation. Figure 1 illustrates the experimental design of the study.

### Physical exercise protocol

The physical exercise routine consisted of an 8-week protocol of running on a motorized treadmill built for rodents (Insight Ltda., Brazil). Running was performed at an intensity of 60–70% maximal oxygen uptake (VO_2_), i.e., a treadmill belt velocity of 9–13 m/min, for 30 min. Sessions were conducted 5 days each week at approximately the same time of day during the light time period (21). In the week before the experimental intervention, rats performed daily treadmill running for 10 min to habituate before performing the first VO_2_ test. An indirect VO_2_ running test was performed to determine the individual exercise intensity beginning at a low velocity and increasing by 5 m/min every 3 min until the rat was unable to run. Time to fatigue (min) and the work volume (m/min) were considered as indirect measures of maximum VO_2_ uptake (16,21). During the fourth week of exercise, an additional indirect VO_2_ running test was conducted to adjust the exercise intensity for each rat.

### Ischemia-reperfusion surgery

After 8 weeks of intervention, rats were subjected to the ischemia-reperfusion or sham surgery procedures. The surgery was performed in the morning, under ketamine and xylazine anesthesia, 75 and 10 mg/kg, respectively, given intraperitoneally. Rats were placed on a heating pad, the neck was shaved, and a midline incision was performed. The muscles and trachea were separated; the common carotid arteries were freed from the adventitial sheath and the vagus nerve, and carefully separated prior to occlusion (22). The temporary occlusion of the common carotid arteries lasted 30 min and was performed using a vascular clip. When restoration of blood flow in the carotid arteries was confirmed by careful observation by an experienced researcher, the neck skin incision was then closed and sutured. Body temperature was maintained during surgery, and until the rat awoke, using a heating pad. After awakening, rats were returned to their cages. Sham-operated rats underwent the same surgical procedure without application of the vascular clip.

### Neuromotor tasks

#### Open-field test

To analyze exploratory behavior, each rat was placed in the left quadrant of a 50×50×39 cm open-field arena consisting of a wooden panel painted white and a front wall of transparent glass. Black lines were drawn on the floor to divide it into 12 equal quadrants. The number of crossings and rearings, as measures for locomotor and exploratory activity, respectively, were monitored for 5 min (23).

#### Rotarod test

Rats were first trained to walk on the rotarod (Insight), which was 5×8×20 cm in diameter, width and height, respectively, at a constant rotational speed of 16 rpm for 1 min. During training, rats were placed back on the rod each time they fell off until the session was completed. At 24 h after training, rats were tested on the rotarod at a constant speed of 20 rpm. Each test consisted of 5 trials lasting 60 s each. The time at which the rat fell off the rotarod and the number of falls were recorded. Rats that fell more than five times were excluded from the experiment and were returned to their cages (24).

#### Nociception evaluation

Nociception was measured using the tail-flick test (25), in which pain was induced by applying an infrared light to the rat's tail 5 cm from the tip. Reaction time (tail-flick latency) was measured as the interval between placing the tail on the infrared light source and its voluntary withdrawal (25).

#### Striatum oxidative status assessment

For tissue preparation, rats were euthanized 24 h after the behavioral experiments were completed. The brain was removed, and the striatum was quickly dissected and homogenized in 50 mM Tris HCl, pH 7.4 (1/10, w/v). The tissue samples were centrifuged at 2400 *g* for 20 min, and supernatants (S1) were used for subsequent assays.

#### ROS

ROS content was assayed spectrofluorimetrically (Shimadzu model RF-5301PC, Japan) using 2',7'-dichlorofluorescein diacetate (DCFH-DA) as a probe. S1 samples were incubated in the dark with 5 µL DCFH-DA (1 mM) and intracellular ROS were detected by the oxidation of DCHF-DA to fluorescent dichlorofluorescein (DCF). DCF fluorescence intensity was recorded at 520 nm (480 nm excitation) 30 min after the addition of DCFH-DA to the medium. Results are reported as AU (arbitrary units).

#### Lipoperoxidation assay

Lipoperoxidation activity was assayed by the formation of thiobarbituric acid reactive substance (TBARS) (26). One aliquot of S1 was incubated with a 0.8% thiobarbituric acid solution in acetic acid buffer (pH 3.2) and 8% sodium dodecyl sulfate at 95°C for 2 h, and the color reaction was measured at 532 nm. Results are reported as nmol of malondialdehyde (MDA) per mg protein.

#### Antioxidant markers

Catalase (CAT) activity was determined spectrophotometrically at 240 nm (27) by monitoring H_2_O_2_ consumption in the presence of a 20 μL sample (S1). Enzyme activity is reported in units (1 U=1 μmol H_2_O_2_ decomposed/min, at pH 7 and 25°C). Glutathione (GSH) levels were determined fluorometrically (28). An aliquot of the homogenized sample was mixed (1:1) with perchloric acid (HClO_4_) and centrifuged at 3000 *g* for 10 min. After centrifugation, the protein pellet was discarded and free-SH groups were determined in the clear supernatant. An aliquot of supernatant was incubated with ortho-phthalaldehyde, and fluorescence was measured at an excitation wavelength of 350 nm and an emission wavelength of 420 nm. Results are reported as nmol/g of tissue. Superoxide dismutase (SOD) activity was measured as previously described (29) by inhibition of the auto-oxidation of epinephrine to adrenochrome. The color reaction was monitored at 480 nm. One enzymatic unit (1 IU) was defined as the amount of enzyme necessary to inhibit the auto-oxidation rate by 50% at 26°C.

### Statistical analysis

The normality of the data distributions was verified using the Shapiro-Wilk test. Open-field and rotarod test results were compared between groups using the Kruskal-Wallis and Dunn's *post hoc* tests. The Mann-Whitney test was used for further comparisons between pairs of groups. One-way analysis of variance (ANOVA) and independent *t*-tests were used to compare between-group differences in tail flick, ROS, TBARS, CAT, GSH, and SOD data. In all cases, statistical significance was set at P<0.05.

## Results

### Neuromotor results

**Figure 2:**
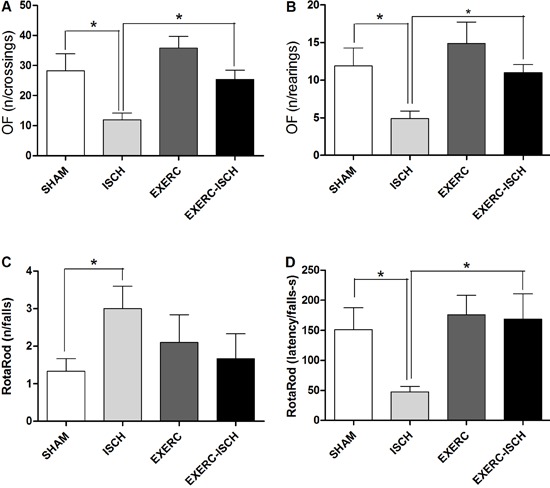
Transient global ischemia-reperfusion led to motor alterations and physical exercise prevented such alterations. *A* and *B*, Results of the open-field (OF) test. The number of crossings are shown in *A* and the number of rearings are shown in *B*. *C* and *D*, Results of the rotarod test. The number of falls are shown in *C* and the latency of the first fall (in seconds) is shown in *D*. Data are reported as means±SD for n=10 rats/group. SHAM: rats submitted to surgery without arterial occlusion; ISCH: rats submitted to ischemia-reperfusion surgery; EXERC: rats submitted to physical exercise before surgery without arterial occlusion; EXERC-ISCH: rats submitted to physical exercise before ischemia-reperfusion surgery. *P<0.05 (Kruskal-Wallis test followed by Mann-Whitney test).

Results of the open-field test (P=0.001 for crossings; P=0.01 for rearings; Kruskal-Wallis) and the rotarod test (P=0.03; Kruskal-Wallis) revealed significant differences between the groups. Neuromotor deficits were observed in the rats subjected to ischemia-reperfusion surgery. In the open-field test, impaired performance of crossings (P=0.048; Figure 2A) and rearings (P=0.024; Figure 2B) were observed in the ischemia-reperfusion group compared with the sham group. Crossings (P=0.260; Figure 2A) and rearings (P=0.480; Figure 2B) were similar in the physical exercise and sham groups. Physical exercise minimized the deficits resulting from ischemia-reperfusion, as shown by the crossings (P=0.003; Figure 2A) and rearings (P=0.004; Figure 2B) data.

Rotarod test performance was impaired in the ischemia-reperfusion group compared with the sham group as shown by the number of falls (P=0.034; Figure 2C) and the times at which the rats fell off the rotarod (P=0.038; Figure 2D). Exercise *per se* did not improve performance on the rotarod test, as the number of falls (P=0.700; Figure 2C) and the times at which the rats fell off the rotarod observed in the physical exercise and sham groups were similar (P=0.650; Figure 2D). Exercise did not decrease the number of falls among rats in the ischemia-reperfusion group (P=0.140; Figure 2C), but it significantly increased the latency to the first fall (P=0.020; Figure 2D).

### Nociception

**Figure 3:**
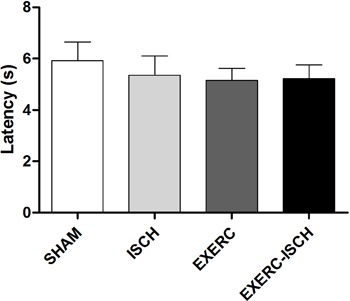
Transient global ischemia-reperfusion did not alter pain sensitivity measured by the tail-flick test. Data are reported as means±SD for n=10 rats/group. SHAM: rats submitted to surgery without arterial occlusion; ISCH: rats submitted to ischemia-reperfusion surgery; EXERC: rats submitted to physical exercise before surgery without arterial occlusion; EXERC-ISCH: rats submitted to physical exercise before ischemia-reperfusion surgery.

Pain sensitivity was similar among rats in the four experimental groups (P=0.800; one-way ANOVA; Figure 3).

### Oxidative status of the striatum

**Figure 4:**
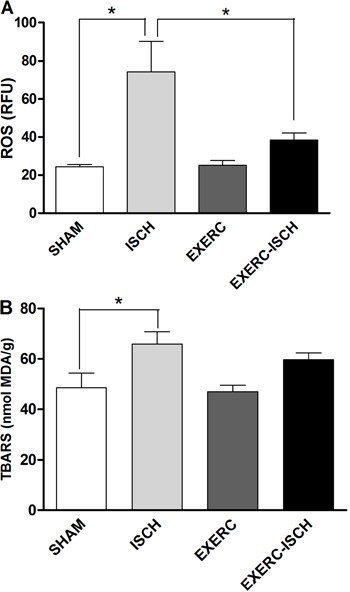
Transient global ischemia-reperfusion promotes an increase of reactive oxygen species and oxidative damage (lipoperoxidation) in the striatum, and physical exercise partially prevents these alterations. *A*, Reactive oxygen species (ROS) levels in the striatum and *B,* lipoperoxidation assessed by TBARS. Data are reported as means±SD. SHAM: rats submitted to surgery without arterial occlusion; ISCH: rats submitted to ischemia-reperfusion surgery; EXERC: rats submitted to physical exercise before surgery without arterial occlusion; EXERC-ISCH: rats submitted to physical exercise before ischemia-reperfusion surgery. *P<0.05 (one-way ANOVA; *t*-test).

Increased oxidative stress status in the striatum was observed, as shown by the increase in ROS levels without any change in antioxidant markers after ischemia-reperfusion. Physical exercise partially reversed this condition. Ischemia-reperfusion increased ROS (P=0.020; Figure 4A) and TBARS (P=0.040; Figure 4B) in the striatum, and physical exercise reduced the increase in ROS levels (P=0.090; Figure 4A) but not the increase in TBARS (P=0.250; Figure 4B).

**Figure 5:**
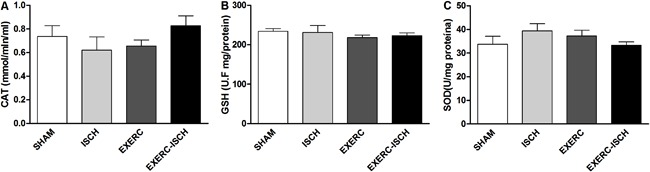
Transient global ischemia-reperfusion and physical exercise did not alter antioxidant markers. *A*, catalase activity (CAT), *B*, glutathione (GSH) levels, and *C*, superoxide dismutase activity (SOD) in the four groups. Data are reported as means±SD. SHAM: rats submitted to surgery without arterial occlusion; ISCH: rats submitted to ischemia-reperfusion surgery; EXERC: rats submitted to physical exercise before surgery without arterial occlusion; EXERC-ISCH: rats submitted to physical exercise before ischemia-reperfusion surgery. There were no significant differences among groups (one-way ANOVA; *t*-test).

No significant differences in the activities of the antioxidant markers that were assayed were observed among the groups (CAT, P=0.390; GSH, P=0.700; SOD, P=0.340; one-way ANOVA; Figure 5).

## Discussion

We assessed the neuroprotective role of physical exercise on striatal oxidative balance and motor impairments resulting from ischemia-reperfusion injury in rats. Our results indicated that ischemia-reperfusion led to significant neuromotor impairments without changing nociception. Animals subjected to ischemia-reperfusion also experienced oxidative stress resulting in oxidative imbalance in the striatum. The damage observed in the striatum is consistent with the neuromotor deficits observed in the ischemia-reperfusion group (9). In this study, neuromotor impairment was demonstrated by the results of the rotarod and open-field tests.

Among our main findings is the capability of physical exercise to protect, although partially, against impairments resulting from an ischemia-reperfusion insult. Physical exercise reversed the impairments in the rotarod test (i.e., time to the first fall) and open-field test (i.e., crossings and rearings) performance observed in the ischemia-reperfusion group. Ischemia-reperfusion is known to induce the deficits in motor development and balance that are measured by the rotarod test (30) and has also been associated with lower neuronal density and area in the striatum after the ischemia-reperfusion injury (30). A neuroprotective role of exercise performed for 14 days before ischemia has been reported in rats, with similar effects in rats trained after ischemia (5).

Nociception was not affected by the ischemic event, which may be explained by the fact that nociception does not depend on striatal activity, but by nociception-specific cortical regions, areas were not evaluated in this study. Those cortical regions are also known to be less sensitive to ischemic events than the hippocampus and striatum are (31). The lack of change in nociception supports a model in which the motor impairments we observed resulted from damage to the striatum.

The motor impairments observed here most likely resulted from the ischemic event that the rats experienced (16). Neuronal degeneration induced by ischemia-reperfusion is associated with conditions of oxidative stress resulting from high levels of fatty acids in the brain (6). The striatum is one of the brain regions most affected by oxidative stress in ischemia-reperfusion and its relatively high densities of GABA receptors and glutamatergic neurons may be related to this neurotoxicity (12). Oxidative stress plays a major role in various pathological conditions, and it may occur in the striatum during aging (17), chronic unpredictable stress situations (32), neurodegenerative diseases such as Alzheimer's and Parkinson's diseases (18,33), and also after strokes (16). Oxidative imbalance in the striatum is related to the loss of dopaminergic neurons and neurotoxicity (12,34) and to damage to DNA/RNA, lipids and proteins, resulting in altered cellular and molecular function and increased cell death (6,8,35,36).

The motor impairments observed here appear to be related to increased levels of ROS and lipoperoxidation in the striatum, leading to oxidative imbalance in this brain region (34). We found that exercise was effective for avoiding motor impairments and that it decreased ROS levels but not lipoperoxidation activity. A recent evaluation of the neuroprotective role of exercise in ischemia-reperfusion injury reported similar results for lipoperoxidation (16). The effects of exercise may be mediated by mitochondrial biogenesis; edema reduction, which would improve blood flow in the ischemic region; and the attenuation of acute neurotoxicity, which would facilitate the reorganization of the injured brain tissue (12,13,37,38). As expected, levels of antioxidant enzymes (CAT and SOD) and GSH, a key non-enzymatic antioxidant, were not changed by exercise (37). Cechetti et al. (16) reported similar results in the cortex and hippocampus.

Understanding the mechanisms of brain injury, the brain’s defense responses, and the adaptations in response to long-term exercise is important for improving the strategies for rehabilitation after ischemic events. Our research supports a model in which physical exercise reverses deficits in locomotor behavior and striatal oxidative balance but does not improve antioxidant status. We found that a relatively short period of physical conditioning benefited animals subjected to ischemia-reperfusion surgery.

In summary, our results demonstrate that physical exercise performed during 8 weeks before ischemia-reperfusion was effective to avoid or minimize motor deficits and oxidative stress conditions in the striatum. In this animal model, exercise was neuroprotective, attenuating the severity of ischemia-reperfusion sequelae.
